# Vanadium-doped phosphomolybdic acids as catalysts for geraniol oxidation with hydrogen peroxide†

**DOI:** 10.1039/d2ra01258h

**Published:** 2022-04-19

**Authors:** Márcio José da Silva, Jonh Alexander Vergara Torres, Castelo Bandane Vilanculo

**Affiliations:** Marcio Jose da Silva, Chemistry Department, Federal University of Vicosa, University Campus Avenue P.H. Rolfs, Vicosa 36570-000 Minas Gerais State Brazil silvamj2003@ufv.br +55 31 3612 6638; Castelo Bandane Vilanculo, Chemistry Department, Pedagogic University of Mozambique, FCNM, Campus of Lhanguene Av. de Moçambique, Km 1 Maputo Mozambique 4040 castelovilanculo@gmail.com +258 875573337

## Abstract

In this work, vanadium-doped phosphomolybdic acids were evaluated as catalysts in green oxidation routes of terpene alcohols with hydrogen peroxide. A series of phosphomolybdic acids containing a variable load of vanadium cations (*i.e.*, V^5+^ ions) were synthesized, and tested as catalysts in geraniol oxidation, the model molecule selected. All the catalysts were characterized by powder X-ray diffraction, attenuated diffuse reflectance infrared spectroscopy, UV-Vis spectroscopy, thermogravimetric analysis, N_2_ adsorption–desorption isotherms, scanning electronic microscopy, X-ray dispersive spectroscopy, and *n*-butylamine potentiometric titration. Various catalysts were evaluated; phosphomolybdic acids with general formulae H_3+*n*_PMo_12−*n*_V_*n*_O_40_ (*n* = 0, 1, 2 and 3), and common Brønsted acids (*i.e.*, H_2_SO_4_, H_3_PO_4_, and *p*-toluene sulfonic acid). Among them, vanadium monosubstituted phosphomolybdic acid was the most active catalyst and selective toward epoxide. The effect of main reaction variables, such as temperature, load catalyst, and reactant stoichiometry was assessed. Evaluating the effect of substrate, it was verified that only allylic alcohols such as geraniol and nerol were successfully epoxidized, demonstrating that this is a hydroxy group-assisted reaction. The effect of vanadium doping on the physicochemical properties of the phosphomolybdic acid catalysts was evaluated and used to explain their catalytic performance.

## Abstract 2

The vanadium-doped phosphomolybdic acid (H_4_PMo_11_VO_40_) was the most active and selective heteropoly catalyst in one-pot oxidative esterification of benzaldehyde with hydrogen peroxide.

## Graphical abstract

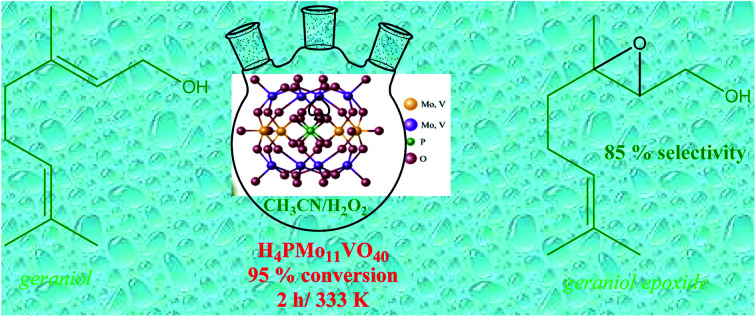

## 1 Introduction

Terpene alcohols oxidation is a key reaction for the fine chemicals industry because it can produce valuable compounds useful as fragrance ingredients, pharmacies, and building blocks in the synthesis of drugs and agrochemicals.^1–6^ These reactions become more attractive when employing green oxidants such as hydrogen peroxide, which generates water as a by-product, is non-flammable and is an easy to handle reactant.^7,8^ Nonetheless, hydrogen peroxide always requires a metal catalyst to be activated.^9,10^

Several transition metal catalysts have been used in oxidations of terpene alcohols with hydrogen peroxide: niobium,^11^ tungsten,^12^ titanium,^13^ and mixed metal oxides^14,15^ are only some examples. Nonetheless, there is a class of compounds known as heteropolyacids (*i.e.*, HPAs) that have received considerable attention.^16^ These polyoxometalates (POMs) are well-defined metal–oxygen clusters that contain oxygen atom bridges linking transition metal atoms with high oxidation states, such as molybdenum, or tungsten.^17,18^

Keggin HPAs have been widely explored catalysts mainly due to their strong Brønsted acidity, high thermal stability, and easily modifiable chemical or physical properties such as their redox potential and solubility.^19,20^ They are solid polyoxometalates with a very strong Brønsted acidity, an advantage when they are used in acid-catalyzed reactions. However, it can be a drawback in the oxidation reactions of terpene alcohols, which are unsaturated substrates that can be oligomerized in the presence of HPAs.^21,22^

However, due to their highly versatile structure, Keggin HPAs can be modified aiming to adjust their physicochemical properties such as solubility, surface area, the strength of acidity and redox potential.^23,24^ The protons exchange by Lewis acid metal cations likewise Sn^2+^, Al^3+^, or Fe^3+^ can significantly improve the activity of Keggin HPAs in acid-catalyzed reactions.^25–28^ In addition, the replacement of protons by cations with a large ionic radius (*i.e.*, cesium, potassium, or ammonium) can modify the solubility of the Keggin HPAs making them insoluble in polar solvents.^29–31^ This alteration kept intact the Keggin anion, which is the primary structure of these catalysts. Keggin HPAs have been also used as solid supported catalysts.^18,32,33^ However, the conversion of Keggin HPAs to insoluble salts has the advantage of avoiding typical problems of solid-supported catalysts, for instance, the leaching of the active phase.^33^ When an insoluble salt is used as a catalyst, there is only a component, that is the salt itself, different from solid-supported catalysts which are constituted by a dopant and by the support.

Another change that can enhance the activity of Keggin HPAs in oxidation reactions is the removal of one MO unit (M = W, Mo) from heteropolyanion, which creates a lacunar catalyst whose redox potential makes them efficient catalysts to be used in oxidation reactions of terpene compounds.^34–37^ On the other hand, the vacancy in the anion of these lacunar catalysts can be filled with a transition metal cation, giving to them a high activity in oxidation reactions with hydrogen peroxide.^38–41^

Besides these structural modifications, another approach frequently adopted mainly when the phosphomolybdic acid is used as a catalyst in oxidation reactions is substituting one or more Mo^6+^ cations with V^5+^ ions.^42–44^ The literature describes that such modification accelerates the redox steps where these catalysts are involved, enhancing the catalytic performance.^45,46^ Most of these vanadium-doped phosphomolybdic acids have been also altered by the protons exchange by cations that have a large ionic radius. These salts become then insoluble and consequently can be used as heterogeneous catalysts. However, they have been used mainly in aerobic oxidation reactions of linear hydrocarbons (*i.e.*, ethane, propane, butane)^47–49^ and aromatic alcohols,^50^ and isobutane.^51–53^ The vanadium–molybdenum heteropolyacids are also used as solid-supported catalysts, however, similarly to their salts, their application has been restricted to oxidations with molecular oxygen.^54,55^ There are scarce examples of the use of vanadium-doped phosphomolybdic acid catalysts in oxidations with hydrogen peroxide.^56,57^

Inspired by these findings, we have decided to investigate the impact of vanadium on the activity of the phosphomolybdic acid catalyst in oxidation reactions with hydrogen peroxide. Thus, sodium salts of vanadium-doped phosphomolybdic acid were synthesized and evaluated in two distinct oxidation reactions with hydrogen peroxide: oxidative esterification of benzaldehyde, and oxidation of terpene alcohols.^58,59^ In both processes, vanadium monosubstituted sodium phosphomolybdate salt was the most effective catalyst. In parallel work, we have found that pristine phosphomolybdic acid itself can efficiently catalyze the oxidation of nerol, allylic terpene alcohol.^60^

In this work, the main objective was to assess how vanadium doping impacts the activity of phosphomolybdic acid catalysts. Therefore, phosphomolybdic acids with different vanadium content (H_3+*n*_PMo_12−*n*_V_*n*_O_40_ (*n* = 0, 1, 2 and 3)) were synthesized, characterized, and evaluated as catalysts in oxidation reactions of geraniol with hydrogen peroxide. Other Brønsted acids (*i.e.*, sulfuric and *p*-toluene sulfonic acids) were also evaluated and compared to the vanadium doped catalysts. The impacts of main reaction variables were assessed such as temperature, catalyst load, molar ratio oxidant: substrate, and reaction time. The redox potential and the strength of acidity were the most impacted characteristics by the vanadium doping and allow to improve the performance of these catalysts.

## 2 Experimental section

### 2.1. Chemicals

All solvents and chemicals were acquired from commercial sources. Geraniol, nerol, geraniol, β-citronellol and linalool were all Sigma-Aldrich (99 wt%), sodium carbonate (99.5 wt%) and diethyl ether (99.8 wt%) were Proquímicos. Na_2_HPO_4_ (99 wt%) was purchased from Riedel de Haen. Hydrate phosphomolybdic acid (99 wt%) was acquired from Sigma-Aldrich, as well as the other synthesis precursors, V_2_O_5_ (99.6 wt%), MoO_3_ (99.5 wt%), H_3_PO_4_ (85 wt%), NaVO_3_ (98 wt%), Na_2_MoO_4_ (≥98 wt%), CH_3_CN (99 wt%). Aqueous hydrogen peroxide (35 wt%) was acquired from Alphatec. H_2_SO_4_ (95–98 wt%) and H_3_PO_4_ (85 wt%) were purchased from Dinâmica.

### 2.2. Synthesis of the H_4_PMo_11_VO_40_

The H_4_PMo_11_VO_40_ acid was synthesized according to the literature.^52^ Typically, the metal oxides MoO_3_ (15.8 g; 110 mmol) and V_2_O_5_ (0.9 g; 4.9 mmol) were dissolved in 350 mL of deionized water and heated to boiling. Then, 1.2 g (1.2 mmol) of H_3_PO_4_ was added and the resulting mixture was refluxed for a 6 h period. A clear solution was obtained cooling to room temperature. The solid acid H_4_PMo_11_VO_40_ was obtained after evaporation of the solvent and then recrystallized, and subsequent drying at 373 K/5 hours.

### 2.3. Synthesis of the H_5_PMo_10_V_2_O_40_

The catalyst was prepared according to the original^61^ and adapted literature.^62^ Firstly, an aqueous solution (100 mL) containing Na_2_HPO_4_ (7.1 g; 50 mmol) was added to a hot aqueous solution (100 mL) containing 24.4 g (0.133 mol) of Na_3_VO_4_. After it has been cooled to room temperature, concentrated H_2_SO_4_ (5 mL) was slowly added, and the solution developed a red colour. Afterwards, Na_2_MoO_4_ 2·H_2_O (121 g; 590 mmol) was solved in water (200 mL) and added to the red solution under vigorous stirring. Once more, concentrated H_2_SO_4_ (85 mL) was slowly added, and the hot solution was cooled to room temperature. Finally, the solution was extracted with diethyl ether, which was vapoured under airflow giving the solid acid H_5_PMo_10_V_2_O_40_. The resulting solid was dried at 373 K/5 h.

### 2.4. Synthesis of the H_6_PMo_9_V_3_O_40_

After adjusting the stoichiometric amount of reactants, the H_6_PMo_9_V_3_O_40_ acid was prepared following a similar procedure to the one described in Section 2.3. However, in this case, the resultant solution was a cherry red colour. After extraction with diethyl ether, it was vaporized and recrystallized in water.

### 2.5. Catalysts characterization

FT-IR/ATR spectra were recorded on Varian 660-IR spectrometer (400–4000 cm^−1^). UV-vis spectra were obtained in CH_3_CN solutions in quartz cuvettes at room temperature with an AJX-6100 PC double bean Micronal spectrometer, fitted with tungsten and deuterium lamps. Solutions of 0.002 molar concentration were chosen since this was the concentration used in most of the catalytic runs.

XRD patterns of the vanadium-doped phosphomolybdic acids were analyzed using an X-rays diffraction system model D8-Discover Bruker using Ni filtered Cu-Kα radiation (*λ* = 1.5418 Å), working at 40 kV and 40 mA, with a counting time of 1.0 s in the diffraction angle (2*θ*) ranging from 5 to 80°.

The porosity and surface area of catalysts were studied by N_2_ adsorption/desorption isotherms in a NOVA 1200e High Speed, Automated Surface Area and Pore Size Analyzer Quantachrome Instruments. Before the analyses, the samples were 1 h degassed. Applying the Brunauer–Emmett–Teller equation (BET) to the N_2_ adsorption–desorption isotherms provided the surface area of the HPA catalysts. To characterize the surface of the solid acids, thin sections were selected and metalized with carbon for analysis with scanning electron microscopy (SEM) and dispersive energy X-rays spectroscopy (EDS) using a JEOL JSM 6010LA SEM.

The acidity strength of catalysts was estimated by potentiometric titration, as described by Pizzio *et al.*^63^ The electrode potential variation was measured with a potentiometer (*i.e.*, Bel, model W3B). Typically, 50 mg of heteropolyacid was dissolved in CH_3_CN and then titrated with the *n*-butylamine solution in toluene (0.05 mol L^−1^).

### 2.6. Identification of main reaction products

The main reaction products were identified in a Shimadzu GC-2010 gas chromatography coupled with a MS-QP 2010 mass spectrometer (*i.e.*, electronic impact 70 eV, scanning range of *m*/*z* 50–450). The spectroscopic characterization and identification of all the products were previously published.^35^ Since the products are not commercially available, they were previously synthesized, purified through silica column chromatography and used as chromatographic standards.

### 2.7. Catalytic tests

Catalytic runs were carried out in a three-necked glass flask (25 mL), fitted with a reflux condenser and sampling septum, under a magnetic stirrer. Geraniol was the model molecule. Usually, geraniol (2.75 mmol) and aqueous H_2_O_2_ (34 wt%) were solved in CH_3_CN (10 mL) and heated to 333 K. The addition of the acid catalyst (*ca.* 0.66 mol%) started the reaction.

The reaction progress was followed for 8 h, regularly collecting samples and analyzing them in GC equipment (Shimadzu 2010, FID), fitted with a Rtx®-Wax, capillary column (30 m length, 0.25 mm i. d., 0.25 mm film thickness). The temperature profile used in gas chromatography analyses was 80 °C (3 min), heating rate (10 °C min^−1^) until 240 °C. Injector and detector temperatures were 250 °C and 280 °C respectively.

The main reaction products were identified by GC-MS analyses on a Shimadzu MS-QP 2010 Ultra mass spectrometer instrument, coupled to Shimadzu 2010 GC (Tokyo, Japan), with He as the carrier gas (1.18 mL min^−1^). Column and chromatographic conditions were similar to the GC analyses. The injector and MS ion source temperatures were 250 and 200 °C, respectively. The MS detector operated in the EI mode at 70 eV, with a scanning range of *m*/*z* 0–400.

## 3 Results and discussion

### 3.1. Catalysts characterization

#### 3.1.1. Infrared spectroscopy

**Fig. 1:**
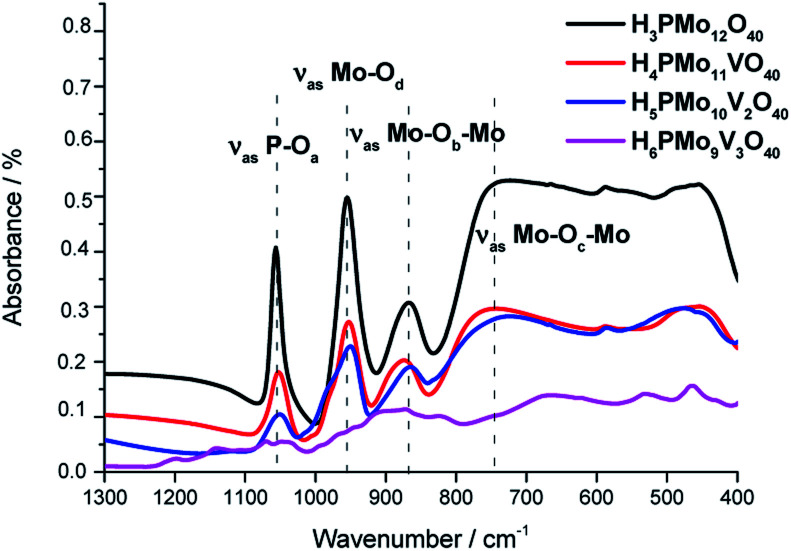
FT-IR/ATR spectra of undoped and vanadium-doped phosphomolybdic acids and phosphomolybdic acid.

The characteristic absorption bands present in the infrared spectrum of Keggin heteropolyanion are noticed in the wavenumber range from 400 to 1700 cm^−1^ (*i.e.*, fingerprint region). Fig. 1 shows the FT-IR spectra of undoped and vanadium doped phosphomolybdic acids. As a reference, dashed lines centred at the main absorption bands were added to it infrared spectra of pristine phosphomolybdic acid.

The absorption band at 1070 was assigned to the stretching vibration of the central oxygen atom *ν*(P–O_a_) bond of the PO_4_ tetrahedron. The other bands were attributed to vibrations of Mo–O bonds where the cation is coordinated to the oxygen atoms in the following positions; peripheral terminal oxygen atom *ν*(Mo–O_d_) bond, the *ν*_ass_(Mo–O_b_–Mo) and *ν*_ass_(Mo–O_c_–Mo) asymmetric stretching of the inter-and intra-octahedral bridges of the trimetallic group, respectively.^64^

The replacement of one or more molybdenum atoms by the vanadium led to a decrease in the heteropolyanion symmetry, which results in a greater number of absorption bands.^65^ It was more noticeable in the infrared spectrum of H_6_PMo_9_V_3_O_40_. Moreover, there was a shift toward lower frequencies of the typical bands of Keggin anion when a higher vanadium load was incorporated, suggesting that the Mo–O–V linkages are weaker than Mo–O–Mo bonds.^66,67^

The absence of a split in the absorption band attributed to the *ν*(P–O_a_) bond vibration in the FT-IR spectra of mono and disubstituted acids (*i.e.*, H_4_PMo_11_VO_40,_ H_5_PMo_10_V_2_O_40_) is evidence that no lacunar anion was formed.^68^ In the infrared spectrum of trisubstituted acid, besides the shift, there was noticed the appearance of shoulders in almost the absorption bands, due to the high vanadium load.^69^

The typical absorption bands of infrared spectra obtained from mono- and disubstituted phosphomolybdic acids were preserved, suggesting that the Keggin structure (*i.e.*, primary structure) was retained after the synthesis. Conversely, the infrared spectrum of H_6_PMo_9_V_3_O_40_ was much different from pristine HPA or other vanadium acids. Therefore, a similar conclusion about the Keggin anion structure was not be done for this acid.^70^ In this case, different from mono and disubstituted acids, vanadium doping drastically modified the profile of the infrared spectrum, probably due to a huge loss of symmetry of anion triggered by the replacement of three molybdenum atoms by vanadium.

#### 3.1.2. UV-visible spectroscopy

The absorption edge in the UV-visible spectra of HPAs measures the energy necessary for “d–d”-type transitions, which occur when one electron is promoted from the Highest Occupied Molecular Orbital (HOMO) to the Lowest Unoccupied Molecular Orbital. In addition, Ligand to Metal Charge Transfer transitions (LMCT), or Metal to Ligand Charge Transfer transitions can be also detected. This information can be useful to evaluate the activity of HPA catalysts in oxidation reactions in the liquid phase.^46^

**Fig. 2:**
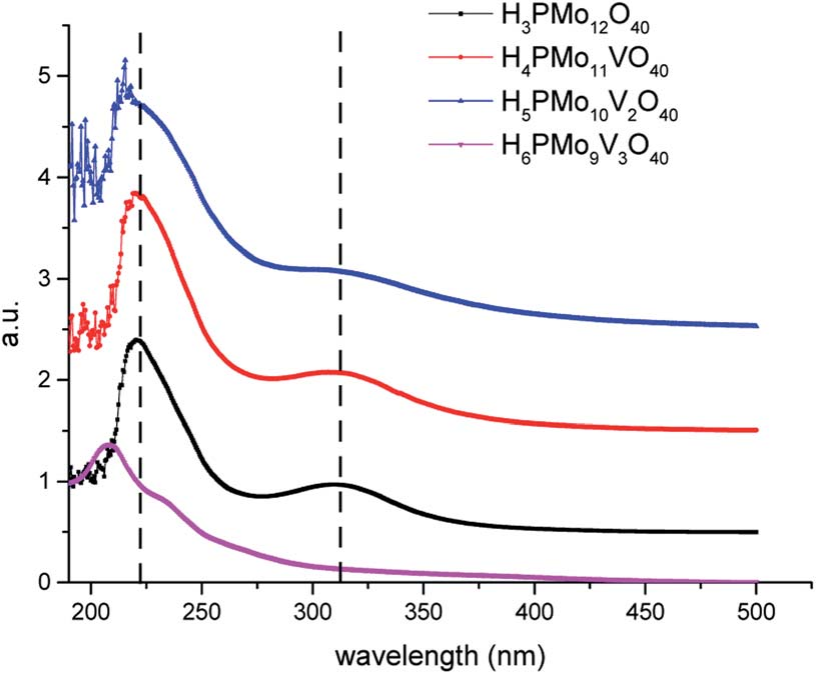
UV-Vis spectra of undoped and doped phosphomolybdic acid catalysts.

Because the HOMO involves mainly terminal oxygen atoms, changes in the framework of HPAs will not affect its energy. Whereas, since the LUMO involves the bridging oxygen atoms and the d-orbitals of the metals, it may be impacted. To verify these effects, UV-Vis spectra of phosphomolybdic acids were recorded (Fig. 2). All the spectra were measured in acetonitrile, except for the H_6_PMo_9_V_3_O_40,_ which was insoluble. Its spectrum was recorded in water.

The most intense absorption band was observed around the 220 nm wavenumber for all the catalysts. These bands were attributed to the octahedrally coordinated Mo^6+^ cations (Fig. 2). An increase in vanadium content shifted the absorption maximum toward a lower wavelength. It can be ascribed to the replacement of one or more Mo cations by V, which reflect changes in the LUMO energy, as well as may also impact the redox properties of the cluster.^71^ Another band can be observed close to 315 nm, however, with a weaker intensity. The Mo^6+^ and V^5+^ cations have d^0^ configuration, and these empty orbitals can receive electrons from ligand oxygen atoms, therefore, this absorption band was assigned to the LMCT transitions (*i.e.*, Mo^6+^ and or V^5+^ to O^2−^).^72^

The effect of vanadium doping led to the shift toward the blue of these absorption bands to a higher-energy region. When the spectra were obtained in CH_3_CN, was possible to perceive the appearance of multiple small bands at a wavelength lower than 210 nm (*ca.* 190–215 nm), which increased with a higher vanadium doping (Fig. 2). The trisubstituted acid was not soluble in CH_3_CN, therefore had its UV-Vis spectrum was obtained in water. Probably due to the different solvents, these bands were more displaced toward lower wavelength in UV spectra of vanadium-trisubstituted-phosphomolybdate.

Barteau *et al.* demonstrated that the level of vanadium doping in the phosphomolybdic acid can be correlated to the absorption edge energies in UV-Vis spectra, as well as to their reduction potential.^42^ Those authors showed that H_4_PMo_11_VO_40_ had higher absorption edge energy and a higher reduction potential than its precursor H_3_PMo_12_O_40_ acid (Table 1). Conversely, they verified that an increase in vanadium doping reduces these two properties.^42^

**Table 1:** Absorption edge energies and reduction potentials of phosphomolybdic catalysts (adapted from ref. 42)

Catalyst	Solution edge (nm)	Edge energy^a^ (eV)	Reduction potential Ag/AgCl (Volts)
H_3_PMo_12_O_40_	468	2.65	−0.082
H_4_PMo_11_VO_40_	526	2.36	0.261
H_5_PMo_10_V_2_O_40_	532	2.33	0.233
H_6_PMo_9_V_3_O_40_	536	2.32	0.168

a Edge energy (*E*) was calculated from the absorption edge wavelength (*k*) by *E* = ℏ*c*/*λ*, where ℏ is Planck's constant and *c* is the speed of light.

#### 3.1.3. Measurement of acidity strength of the undoped and vanadium-doped phosphomolybdic acid catalysts

This technique allows to quantify the total number of acid sites in the catalyst surface, calculate from the point where the curve reaches its plateau, and classify the strength of acidity according to the value of the initial electrode potential; *E*_i_ > 100 mV (very strong sites), 0 < *E*_i_ < 100 mV (strong sites), −100 < *E*_i_ < 0 (weak sites) and *E*_i_ < −100 mV (very weak sites).^63^

**Fig. 3:**
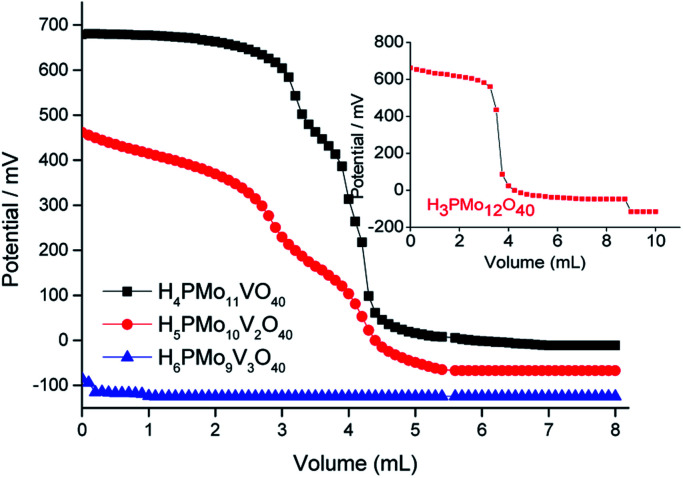
Potentiometric titration curves with *n*-butylamine of pristine phosphomolybdic acid and vanadium-doped derivatives.

The phosphomolybdic acid and their derived mono- and disubstituted presented very strong acid sites. Nonetheless, only the H_4_PMo_11_VO_40_ displays acid sites with a strength higher than H_3_PMo_12_O_40_ (inset in Fig. 3). Vilabrille *et al.* reported that the exchange of Mo^6+^ by V^5+^ into Keggin anion of phosphomolybdic acid weakens the P–O_a_ bond and its interaction with the di-hydronium cations (*i.e.*, H_5_O_2_^+^).^45^ This substitution changes the charge of the distinct oxygen atoms, mainly O_d_ (*i.e.*, terminal oxygen atom), increasing the acidity of the new proton. This specific effect was noticed herein (see titration curves of H_3_PMo_12_O_40_ and H_4_PMo_11_VO_40_, Fig. 3).

On the other hand, an increase in vanadium load leads to a diminishment of the acidity strength of Vanadium-doped phosphomolybdic acids, resulting in a catalyst with the weakest acid sites (*i.e.*, H_6_PMo_9_V_3_O_40_) (Fig. 3). This result was also described by Villabrille *et al.*, which ascribed this effect to the increase in charge of heteropolyanion, with a consequent increase in their number of protons, causing a diminution of the strength of acidity.^45^

**Fig. 4:**
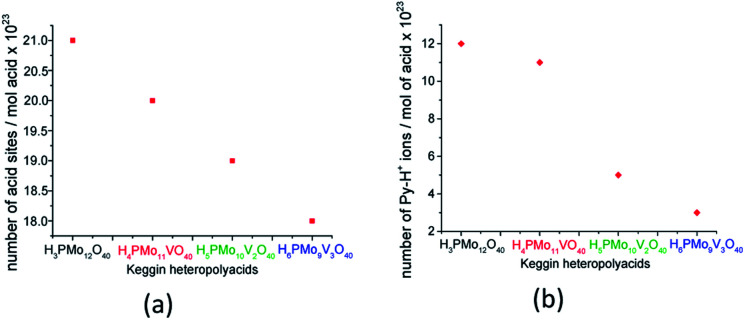
Acid sites concentration was determined from TPD pyridine (a) and the number of Py-H^+^ ions was determined from the infrared intensity band at 1540 cm^−1^ (b) (adapted from ref. 73).

Although useful to compare the acidity of these catalysts, the potentiometric titration does not distinguish Lewis or Brønsted acid sites. Conversely, TPD-pyridine is a technique that solves this question. Serwicka *et al.* performed several TPD-pyridine analyses of a series of H_3+*n*_PMo_12−*n*_V_*n*_O_40_^(3+*n*)−^ (*n* = 0, 1, 2, or 3) acids and verified that an increase in vanadium load reduces the acid sites number and weakens their strength.^73^ In addition, they have found that these catalysts have predominantly Brønsted acid sites, once that pyridine adsorbs as pyridinium cation, at 1540 cm^−1^ wavenumber (Fig. 4).

Serwicka *et al.* demonstrated that the acid sites concentration measured from TPD-pyridine and the number of Py-H^+^ ions obtained from the infrared band intensity at 1540 cm^−1^ are straightly linked (Fig. 4).^73^ It is noteworthy that these two quantities should not be necessarily equal, since that infrared spectroscopy data are exclusively related to one kind of adsorbed pyridine (*i.e.*, pyridinium ion), while TPD measurements can referee also to the adsorbed pyridine as other species on the catalyst surface.^73^

#### 3.1.4. Powder X-rays diffraction patterns of undoped and vanadium-doped phosphomolybdic acid catalysts

**Fig. 5:**
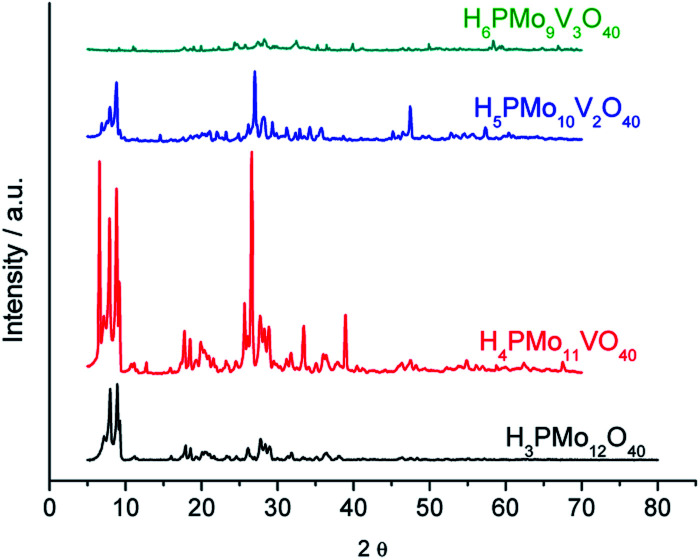
Powder XRD patterns of phosphomolybdic acid and their vanadium doped derivatives.

XRD pattern of Keggin HPAs brings information about the secondary structure, resulting from the arrangement of anions with their counter cations and hydration water molecules, while the infrared spectrum provides data on heteropolyanions (*i.e.*, primary structure).^74^ The presence of metal cations and hydration water molecules may affect the arrangement and symmetry of unitary cells of HPAs.^34^Fig. 5 displays XRD patterns of undoped and vanadium doped phosphomolybdic acids.

The powder X-rays diffraction patterns were taken in the 2*θ* ranges of 5.0° to 70.0°. The most significant peaks in the diffractogram of phosphomolybdic acid were observed at 2*θ* values of (5.0–10.0) °, (18.0–23.0) °, (25.0–30.0) ° and (31–38) ° counts. These values agree with the literature that suggests a body-centred cubic crystalline structure for this acid, and triclinic T for the H_4_PVMo_11_O_40_.^74–76^

A greater vanadium load resulted in higher crystallinity, preserving the main peaks mainly in monosubstituted and disubstituted acids. Nonetheless, new diffractions peaks appeared at greater 2*θ* angles, mainly in the XRD of trisubstituted acid. Shen. *et al.* synthesized a series of H_3+*n*_PMo_12−*n*_V_*n*_O_40_ (*n* = 0, 1, 2 and 3) and their results were like the XRD database of POMs.^77^ Comparing our data with those reported in the literature, we can conclude that the secondary structure remained almost intact after the inclusion of one or two vanadium atoms.^72–77^

#### 3.1.5. Thermal analyses of pristine phosphomolybdic acid and their vanadium doped derivatives

**Fig. 6:**
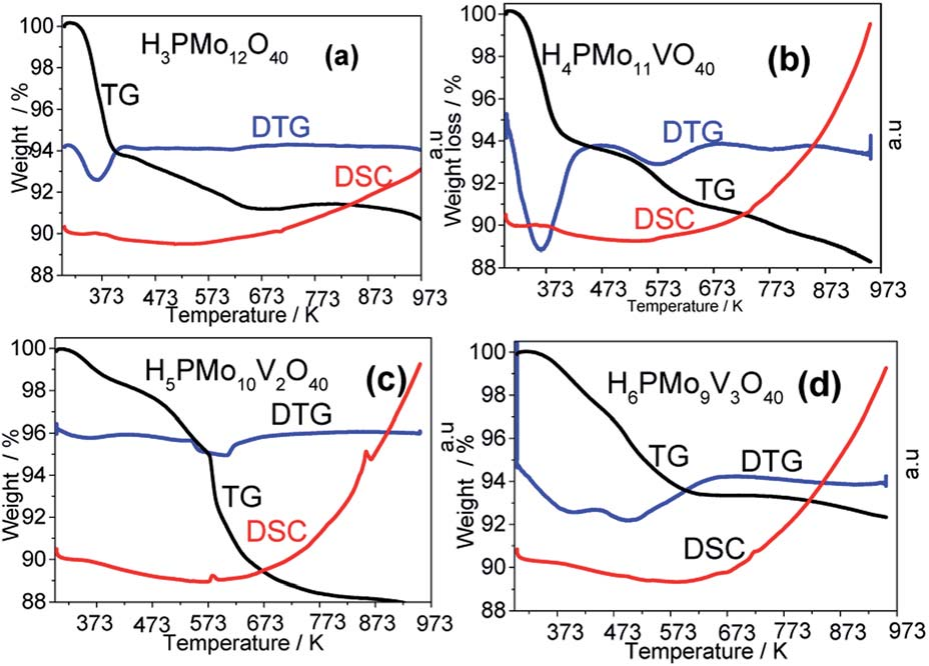
Thermal analysis curves of unsubstituted and vanadium-substituted phosphomolybdic acids.

All the TG curves of vanadium-doped acids had a slower decline than their precursor (Fig. 6), indicating that they were thermally more stable. Although at different temperatures, the losses of weight were ascribed to physisorbed water molecules (*T* < 423 K), crystallization, or constitutional ones (*T* < 473 K). The slower dehydration may be assigned to the stronger entrapment of the water molecules in micropores. The second water loss has occurred between 623 and 823 K is assigned to the deprotonation process.^74^

A quicker loss of water in the TG curve gave a strong endothermic peak in DTG curves, while a more gradual loss gives a less pronounced peak or a broader valley. In Fig. 6a and b (*i.e.*, H_3_PMo_12_O_40_ and H_4_PMo_11_VO_40_, respectively), it was noticed at 373 K. Conversely, when the loss was slower (*i.e.*, Fig. 6a and b, H_5_PMo_10_VO_40_ and H_6_PMo_9_V_3_O_40_, respectively), broad peaks rose around 473 (Fig. 6c) and 473 and 523 K (Fig. 6d).

The literature describes that phosphomolybdic anions are decomposed to oxides (*i.e.*, to P_2_O_5_, MoO_3_ and V_2_O_5_) at temperatures higher than 773 K, due to the collapse of the P–O_a_–Mo framework, which is confirmed by the appearance of an endothermic peak in DSC curves.^78^ However, it was visible only in the DSC curve of H_5_PMo_10_V_2_O_40_ acid, which presented a peak at 863 K and another close to 583 K temperature (Fig. 6c).

To calculate the number of water molecules number present in the acids, we analyzed the DTG curves in Fig. 6 and considered the percentage of weight loss that occurred between 298 to 523 K, which was attributed to the releasing of crystallization, bounded, and structural water molecules.^52^ It was verified that pure acid, mono-, di- and tri-vanadium-doped presented 6, 8, 5 and 6 water moles per mol of Keggin anion (Table 1SM†). Some of the changes observed in XRD patterns of vanadium-doped acids (Fig. 5) can be a consequence of the different hydration levels.^79^

#### 3.1.6. Analyses of porosimetry of undoped and vanadium-doped phosphomolybdic acids

The N_2_ adsorption/desorption isotherms provided the volume, distribution, and pores diameter of vanadium-doped phosphomolybdic acid catalysts and pristine HPA, while the application of the BET method gave the surface area values (Fig. 1SM†).

According to IUPAC rules, the isotherms were classified as being intermediate between type III and V. The slight hysteresis loop in isotherm plots obtained from vanadium-doped phosphomolybdic acids was classified as H-3, evidence that they are a kind of mesoporous material (*ca.* pores size between 5 to 50 nm), with a higher pore size range when the vanadium doping is increased (Fig. 1SM†). It was attributed to the capillarity condensation in mesopores of solid catalysts, a consequence of adsorption on the aggregates of platy particles. In Table 1SM,† the porosimetry properties of vanadium doped phosphomolybdic acids were compared to the pristine acid. Keggin HPAs are solid with a low surface area, however, the vanadium doping increased it from 1.4 (*i.e.*, H_3_PMo_12_O_40_) to 2.7 m^2^ g^−1^ (*i.e.*, H_4_PMo_11_VO_40_). The vanadium doping increased the pores volume of the phosphomolybdic acids, although the diameter has been smaller.

#### 3.1.7. SEM-EDS analyses of undoped and vanadium phosphomolybdic acids

The phosphomolybdic acids with and without vanadium were submitted to SEM-EDS analysis to characterize their surfaces (Fig. 2SM†). The vanadium doping led to a visible reduction in particle size is visible, therefore, it is possible to verify that there was an increase in the surface area of these acids, as demonstrated by the measurements of porosimetry (Table 2SM†). EDS analysis confirmed the percentual elemental composition of vanadium doped phosphomolybdic acids (Fig. 3SM†). No residual element was found (*i.e.*, a sulfuric acid component used in the synthesis), a guarantee that they were adequately purified.

### 3.2. Catalytic tests

#### 3.2.1. Effect of vanadium doping on the conversion and selectivity of phosphomolybdic acid-catalyzed geraniol oxidation

**Fig. 7:**
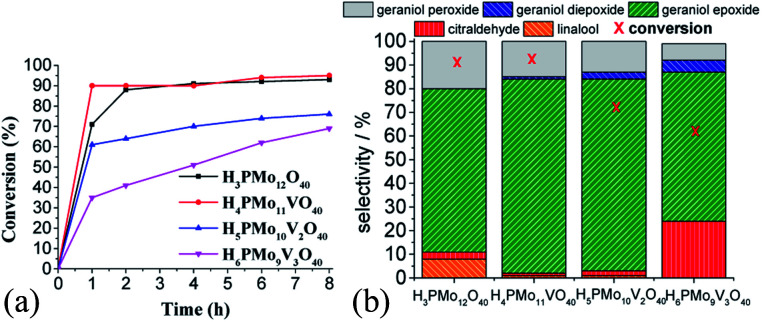
Effect of vanadium doping on the activity of phosphomolybdic acid catalysts on the geraniol oxidation reactions with H_2_O_2_: kinetic curves (a), conversion, and products selectivity after 8 h (b)^*a*^. ^*a*^Reaction conditions: geraniol (2.75 mmol), H_2_O_2_ (2.75 mmol), toluene (internal standard), catalyst (0.66 mol%), temperature (333 K), CH_3_CN (10 mL).

The efficiency of the vanadium-doped phosphomolybdic acids was compared to the original HPA and the main results are summarized in Fig. 7. The reaction conditions were chosen based on the literature.^59^

While the kinetic curves of reactions in the presence of undoped or monosubstituted phosphomolybdic acids have a similar profile, achieving a conversion of 94% within 2 first hours of reaction, those with di- or trisubstituted-vanadium reached 76 and 65% of conversion after 8 h, respectively (Fig. 7a). Table 5SM† shows the rate constant and TON achieved in all of these reactions. These data suggest that H_4_PMo_11_VO_40_ acid was the most active catalyst.

Nevertheless, the highest selectivity toward geraniol epoxide was achieved in the H_4_PMo_11_VO_40_-catalyzed reaction (Fig. 7b). Although epoxide selectivity in presence of disubstituted acid has been slightly lower than monosubstituted, the reaction conversion was visibly lower. Another evidence that a high vanadium load was not beneficial, is that the worst performance was of the trisubstituted acid, either in terms of conversion or selectivity (Fig. 7).

These results can be explained by the reduction potential of vanadium-doped acids; as higher was the reduction potential, as efficient was the catalyst, achieving greater conversion and selectivity toward geraniol epoxide (Table 1).

**Scheme 1:**
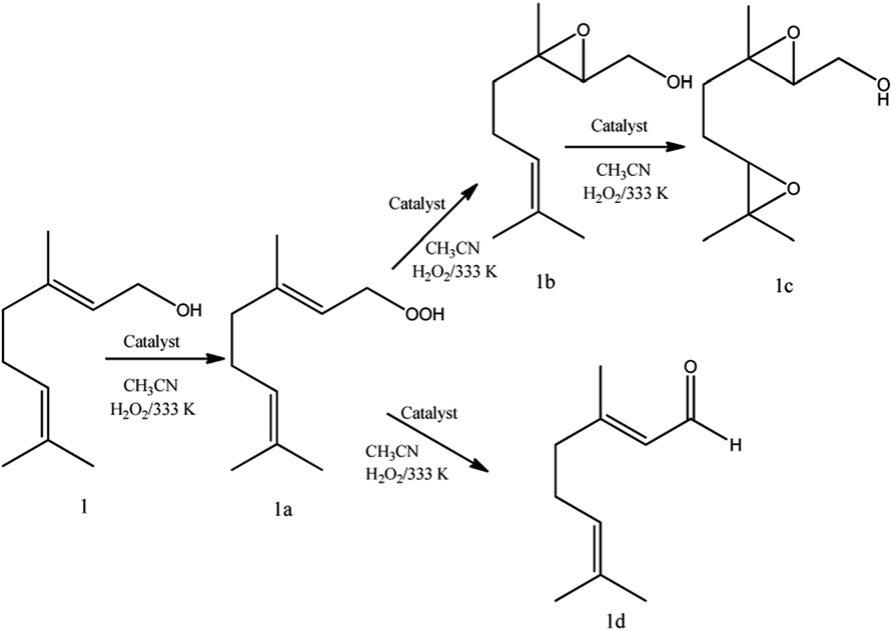
Main products of geraniol oxidation with H_2_O_2_ in the presence of vanadium-doped phosphomolybdic acid catalysts in CH_3_CN solutions.

Regardless of catalyst nature, geraniol epoxide (1b) was always the major product, with geraniol diepoxide (1c) and aldehyde (*i.e.*, citral, 1d) being the secondary products (Scheme 1). The literature describes that peroxide (*i.e.*, geraniol peroxide, 1a) is the most probable intermediate in these reactions, which are non-detected products by GC-FID analysis but were calculated through the mass balance of reactions.^59,60^

**Fig. 8:**
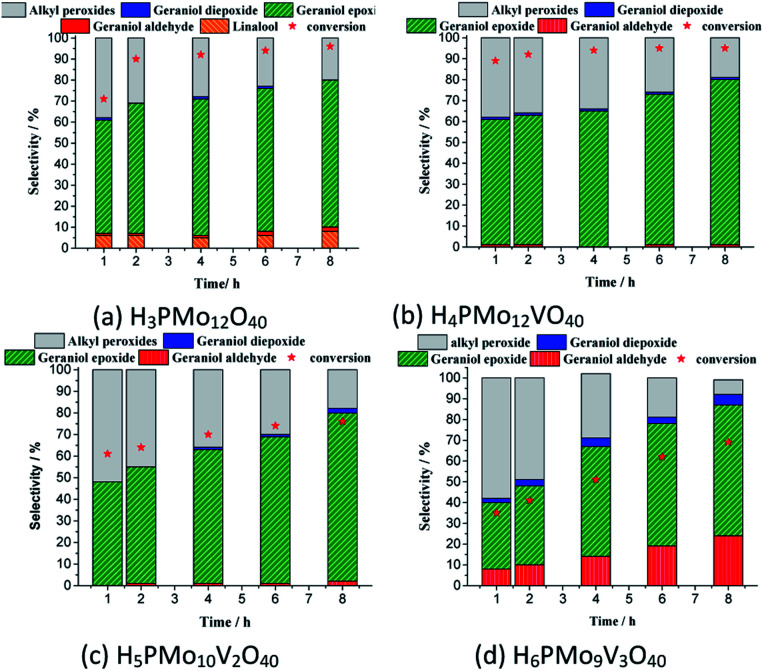
Monitoring of conversion and selectivity of geraniol oxidation reactions by H_2_O_2_ in the presence of phosphomolybdic acid or their Vanadium doped derived^*a*^. ^*a*^Reaction conditions: geraniol (2.75 mmol), H_2_O_2_ (2.75 mmol), toluene (internal standard), temperature (363 K), CH_3_CN (9.3 mL), catalyst load (0.66 mol%).

Although sometimes the reactions of terpene alcohol oxidations can achieve the maximum conversion within the initial period, nonetheless, it was reported that the selectivity can be modified throughout the time reaction.^35^ To check this effect, the reactions were monitored during the runs and displayed the main results in Fig. 8.

In all the runs, regardless of the catalyst, it was demonstrated that throughout the reactions the nerol peroxide is converted to geraniol epoxide. This is evidence that these are consecutive reactions, such as proposed in Scheme 1. Among the catalysts tested, the monosubstituted phosphomolybdic acid was the most efficient achieving the highest conversion and selectivity toward nerol epoxide.

Besides the reduction potential, there is another useful property to explain the behaviour of these acid catalysts in the oxidation reactions of geraniol. Weinstock *et al.* have reported that the Keggin HPAs type (Mo–V–P) possess a strong Brønsted acidity, which is beneficial to the oxidative catalytic transformations of biomass-derived products.^80^ Indeed, literature has described that the formation of the peroxide-metal intermediate between the peroxide and the HPA catalyst (*i.e.*, V, Mo or W HPA) is favoured in the presence of the vanadium atom.^68,81–83^

Even though in the absence of vanadium, the H_3_PMo_12_O_40_-catalyzed reaction achieved a high conversion, however, in this case, it can be attributed to the presence of a higher Brønsted acidity strength, which is translated into better catalytic performance (Fig. 8a).^59^ Recently, we have evaluated the performance of various Brønsted acids in oxidation reactions of nerol with hydrogen peroxide and verified that the activity of H_3_PMo_12_O_40_ was very higher than other HPAs (*i.e.*, H_3_PW_12_O_40_ and H_4_SiW_12_O_40_), and typical Brønsted acids (*i.e.*, H_2_SO_4_ and *p*-toluene sulfonic acid).^60^ Arcoria *et al.* confirmed that an accelerating effect in the epoxidation rate of a simple olefin is triggered by protic species, likely *via* hydrogen bonding of the oxygen atom belonging to the peroxide group in the transition state.^84^

It suggests that both Brønsted (*i.e.*, H_3_O^+^ ions, H_3_PMo_12_O_40_) and Lewis's acid sites (*i.e.*, vanadium sites, H_4_PMo_11_VO_40_), seem to play a key role to epoxidize the geraniol. A reaction mechanism involving a peroxide intermediate bonded to Keggin vanadophosphomolybdate anion was proposed based on these findings.^59^

On the other hand, with a higher vanadium load (*i.e.*, V_2_ and V_3_ catalysts), the catalysts underwent a decline in performance, justified by the increase of energy barrier between HOMO and LUMO orbitals, which difficult the reducibility of these di- or tri-substituted heteropolyanions.^71,85^

Another aspect that distinguished the performance of vanadium doped phosphomolybdic acids was the highest selectivity toward geraniol aldehyde (*i.e.*, citral 1d, Scheme 1) achieved in the H_6_PMo_9_V_3_O_40_-catalyzed reaction. This compound can be obtained by decomposition of peroxide geraniol intermediate.^59^ The literature describes that this composition to a carbonylic compound is more favourable when the catalyst is easily oxidizable, it is, undergone a fast interconversion of one electron (*i.e.*, V^4+^/V^5+^), which is promoted by a high vanadium load.^81–85^ High selectivity to aldehyde was also reached when the sodium salt of this acid (*i.e.*, Na_6_PMo_9_V_3_O_40_) was used in oxidation reactions of nerol.^59^

#### 3.2.2. Effect of H_4_PMo_11_VO_40_ concentration on geraniol oxidation with hydrogen peroxide

**Fig. 9:**
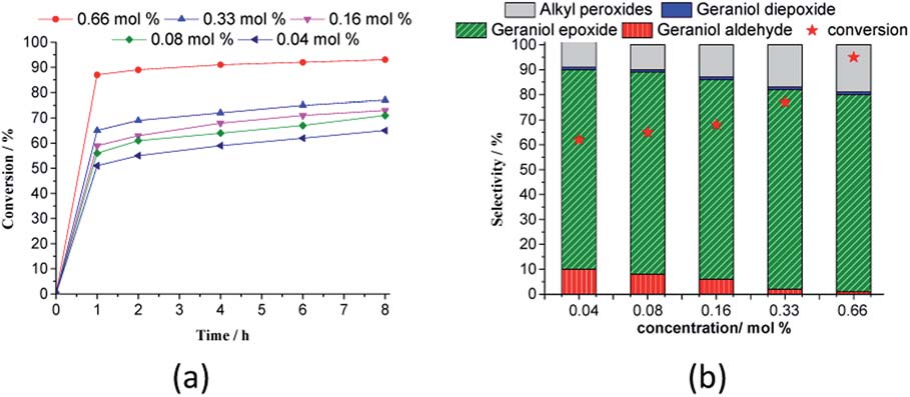
Effect of H_4_PMo_11_VO_40_ catalyst load on the kinetic curves (a), conversion, and products selectivity after 8 h (b) of geraniol oxidation reactions with H_2_O_2_^*a*^. ^*a*^Reaction conditions: nerol (2.75 mmol), H_2_O_2_ (2.75 mmol), toluene (internal standard), temperature (333 K), CH_3_CN (10 mL).

The catalytic activity of H_4_PMo_11_VO_40_ was assessed and the main results are displayed in Fig. 9. An increase in catalyst concentration improved the initial rate of reactions, however, the kinetic curves presented a similar profile, reaching the maximum conversion within the initial period. Table 6SM† shows the rate constant and TON achieved in all of these reactions. These data suggest that H_4_PMo_11_VO_40_ acid was a very active catalyst achieving a TON equal to 1738.

Regardless of the catalyst load, geraniol peroxide remained was always the major product, however, a decrease in the concentration of the H_4_PMoV_1_O_40_ favoured the formation of aldehyde (*i.e.*, citral, Scheme 1).

#### 3.2.3. Effect of temperature on the H_4_PMo_11_VO_40_-catalyzed oxidation of geraniol with H_2_O_2_

**Fig. 10:**
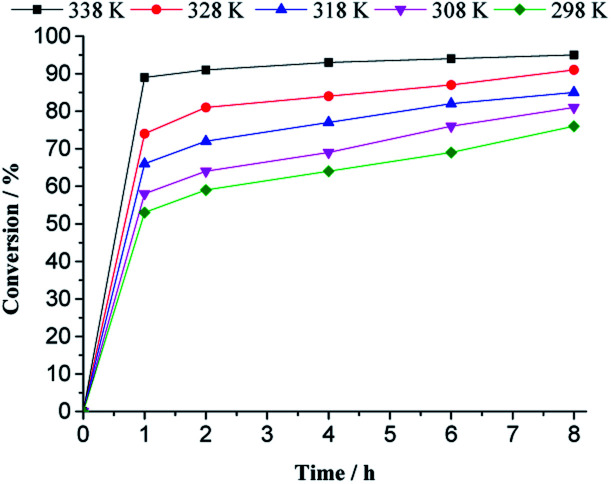
Impacts of temperature on the kinetic curves of H_4_PMo_11_VO_40_-catalyzed geraniol oxidation reaction with H_2_O_2_^*a*^. ^*a*^Reaction conditions: nerol (2.75 mmol), H_2_O_2_ (2.75 mmol), toluene (internal standard), catalyst (0.66 mol%), CH_3_CN (10 mL).

The influence of the temperature on the catalytic performance of the H_4_PMo_11_VO_40_ was investigated and the kinetic curves are presented in Fig. 10.

With a higher temperature, the reactions became faster, due to a higher number of effective collisions, an effect that was much more visible at 333 K. In addition, no significant change was observed in reaction selectivity when the reactions were carried out at different temperatures. In all the runs, geraniol epoxide was always the main product.

#### 3.2.4. Effect of oxidant load on the conversion and selectivity of H_4_PMo_11_VO_40_-catalyzed oxidation with H_2_O_2_

**Fig. 11:**
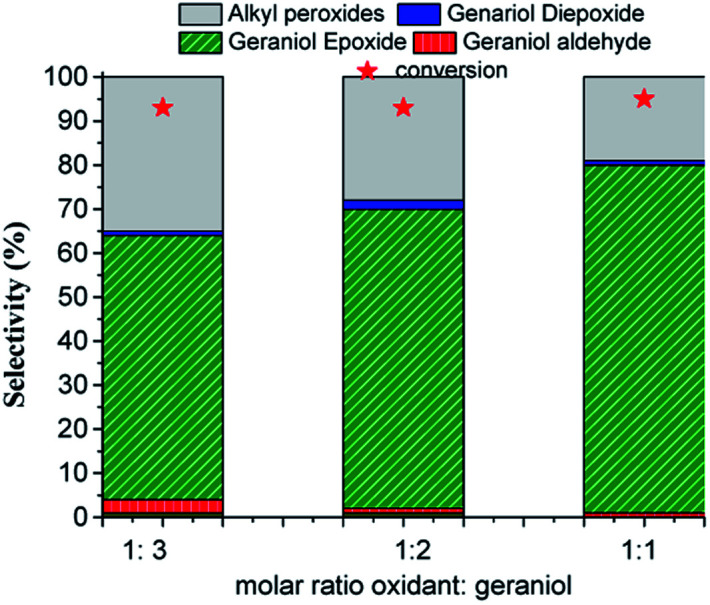
Effect of oxidant substrate molar ratio on the conversion and selectivity of H_4_PMo_11_VO_40_-catalyzed geraniol oxidation reactions with H_2_O_2_^*a*^. ^*a*^Reaction conditions: geraniol (2.75 mmol), catalyst (0.66 mol%), toluene (internal standard), temperature (333 K), CH_3_CN (10 mL).

A greater amount of the oxidant could affect the substrate conversion and the product's selectivity, mainly due to the higher presence of water in the reaction medium. This effect was evaluated the main results are in Fig. 11.

The conversion and the selectivity of products were differently impacted by the increase in oxidant load. While the reactions achieved almost the same conversions, regardless of oxidant load, the selectivity had different behaviour. Although geraniol epoxide was always the main product, an excess of hydrogen peroxide favoured the formation of alkyl peroxides.

#### 3.2.5. H_4_PMo_11_VO_40_-catalyzed oxidation reactions with H_2_O_2_: effect of the substrate

**Scheme 2:**
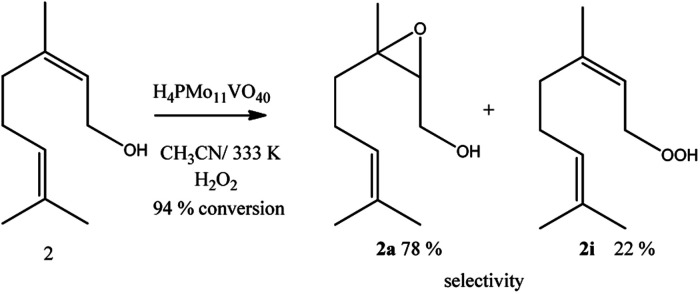
H_4_PMo_11_VO_40_-catalyzed nerol oxidation reactions with H_2_O_2_^*a*^. ^*a*^Reaction conditions: nerol (2.75 mmol), catalyst (0.66 mol%), temperature (333 K), reaction time 8 h, toluene (internal standard), CH_3_CN (10 mL).

**Scheme 3:**
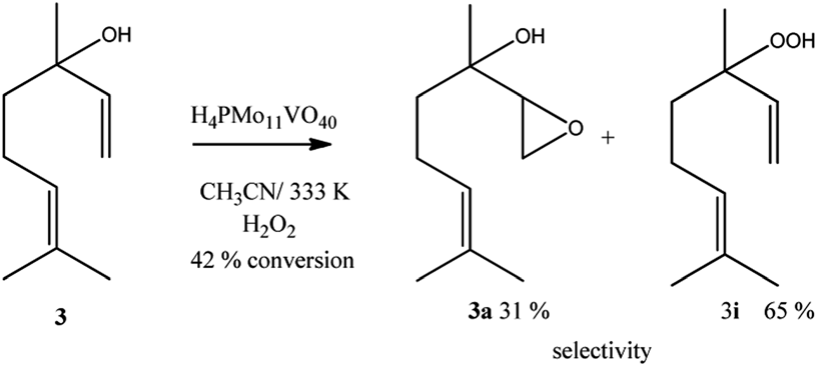
H_4_PMo_11_VO_40_-catalyzed linalool oxidation reactions with H_2_O_2_^*a*^. ^*a*^Reaction conditions: linalool (2.75 mmol), catalyst (0.66 mol%), toluene (internal standard), reaction time (8 h), temperature (333 K), CH_3_CN (10 mL).

**Scheme 4:**
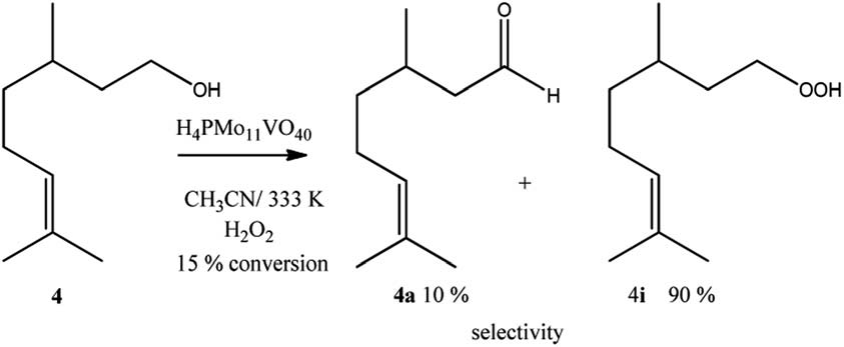
H_4_PMo_11_VO_40_-catalyzed β-citronellol oxidation reactions with H_2_O_2_^*a*^. ^*a*^Reaction conditions: β-citronellol (2.75 mmol), catalyst (0.66 mol%), reaction time (8 h), temperature (333 K), toluene (internal standard), CH_3_CN (10 mL).

To evaluate how electronic and steric effects may affect the conversion and selectivity of the oxidation reactions different alcohols were selected as substrates; besides geraniol, nerol that its geometric isomer (*i.e.*, allylic alcohol), β-citronellol (*i.e.*, primary alcohol), linalool (*i.e.*, tertiary alcohol) were tested in the conditions previously established (Schemes 2–4).

Geraniol and nerol are (*Z*) and (*E*) geometric isomers, respectively. However, the performance of the H_4_PMo_11_VO_40_ catalyst in their oxidation reactions with hydrogen peroxide was almost the same; conversions close to 95% and epoxide selectivity near to 80% were achieved within 2 first hours of reaction (Scheme 2).

Alkyl peroxides, which are probable reaction intermediates, were secondary products in both cases. This same behaviour was noticed in the presence of other catalysts such as tungsten or niobium oxides, or still, metal substituted heteropolyacid catalysts.^17,18,41^ In all these cases, the authors argued that the epoxidation of these alcohols is a hydroxy group assisted reaction.^86^

Linalool, tertiary allylic alcohol was another substrate evaluated. Although it has a terminal double bond, which could be more easily epoxidized, the epoxide selectivity was lower than that achieved (Scheme 3) in oxidations of geraniol or nerol (Schemes 1, 2). The step of oxygen atom transfer from peroxide intermediate to double bond, which is promoted by the presence of vanadium doped catalyst may be less favoured herein due to the steric effects.

Despite the different behaviours, these three terpene alcohols remained with a double bond almost untouched after the oxidation reactions. It suggests that the hydroxy group plays an essential role in these epoxidation reactions. In the absence of an allylic hydroxyl group, epoxidation is overlooked by the oxidation of the hydroxyl bonded carbon to a carbonyl group. It was demonstrated in β-citronellol oxidation, which gave alkyl peroxide and β-citronellal as the main oxidation products (Scheme 4).

Nonetheless, since β-citronellol is a primary alcohol, at these reaction conditions which are limited by the boiling point of the solvent, only a poor conversion was achieved (15%, Scheme 4).

## 4 Conclusions

The epoxidation of terpenic alcohols using phosphomolybdic acids doped with different vanadium loads as catalysts and hydrogen peroxide as oxidant was studied. Using geraniol as the model substrate, the effect of the main reaction variables was assessed. The H_4_PMo_11_VO_40_ acid was the most active catalyst, overcoming the performance of undoped, di- and trisubstituted phosphomolybdic acids. The greatest catalytic activity of monosubstituted acid was assigned to two features; the highest Brønsted acidity and the highest reduction potential. As demonstrated by the results of potentiometric titration, adsorbed Pyr-FT-IR and TPD pyridine (*i.e.*, these two data provided from literature), H_4_PMo_11_VO_40_ is the strongest Brønsted acid. However, the alone Brønsted acidity is not enough to assure the efficiency of the catalyst in these oxidation reactions. A comparison with typical Brønsted acids (*i.e.*, sulfuric and *p*-toluene sulfonic acids) and even with pristine H_3_PMo_12_O_40_, showed that H_4_PMo_11_VO_40_ was the most effective, evidence that vanadium is also required. The doping with one mol of vanadium increases the potential reduction, and accelerates the redox activity of the catalyst; loads higher than one V^5+^ cation/per anion increase the HOMO LUMO energy barrier, compromising the catalyst performance. The reaction scope was extended to other terpene alcohols. We verified that this epoxidation is a hydroxy group assisted reaction; only the double bonds neighbour to hydroxy groups (*i.e.*, geraniol and nerol) were efficiently epoxidized. Conversions higher than 90% and selectivity (80–85) % were achieved after a 1 h reaction with an equimolar amount of hydrogen peroxide and 0.66 mol% of H_4_PMo_11_VO_40_.

## Author contributions

J. A. V. Torres: chemical synthesis and characterization, catalytic experiments. C. B. Vilanculo: investigation, methodology, writing – original. M. J. da Silva: conceptualization, resources, writing – review & editing, visualization, supervision.

## Conflicts of interest

There are no conflicts to declare.

## Supplementary Material

RA-012-D2RA01258H-s001
