# Supplementary material for: Vanadium-doped phosphomolybdic acids as catalysts for geraniol oxidation with hydrogen peroxide
Source: RSC Adv. 2022 Apr 19;12(19):11796–806. doi: 10.1039/d2ra01258h (PMC9016745; doi:10.1039/d2ra01258h)
Supplement: RA-012-D2RA01258H-s001 [file RA-012-D2RA01258H-s001.pdf]

Electronic Supplementary Material (ESI) for RSC Advances.
This journal is © The Royal Society of Chemistry 2022

Impacts of Vanadium doping on the activity of phosphomolybdic acid catalysts in oxidation reactions of geraniol with hydrogen peroxide

MÁRCIO JOSÉ DA SILVA[1*], JONH ALEXANDER VERGARA TORRES[1], AND CASTELO BANDANE VILANCULO[2*]

[1]Chemistry Department, Federal University of Viçosa, Viçosa, Minas Gerais, Brasil. zip-code: 36590-000

[2]Chemistry Department, Pedagogic University of Mozambique, FCNM, Campus de Lhanguene, Av. de Moçambique, km 1, Maputo, C.P.: 4040, Fax: (+258)21401082.

Supplemental material

List of Figures

Figure 1SM. Isotherms of adsorption and desorption of N_2 and volume and diameters porous (inset) of a pure phosphomolybdic acid catalyst and after the Vanadium doping.

Figure 2SM. Scanning electronic microscopy images of undoped and Vanadium-doped phosphomolybdic acids.

Figure 3SM. EDS spectra of undoped and Vanadium-doped phosphomolybdate acids.

Figure 4SM. Typical chromatogram of oxidation reaction with hydrogen peroxide

Figure 5SM1: Fragmentogram of the geraniol epoxide

Figure 6SM1: Fragmentogram of the geraniol diepoxide

Figure 7SM1: Fragmentogram of the nerol epoxide

List of Tables

Table 1SM. Porosimetry characteristics of pure and Vanadium doped-Sodium phosphomolybdate salts[a]

Table 2SM. Hydration water number per mol of catalyst determined through thermal analysis.

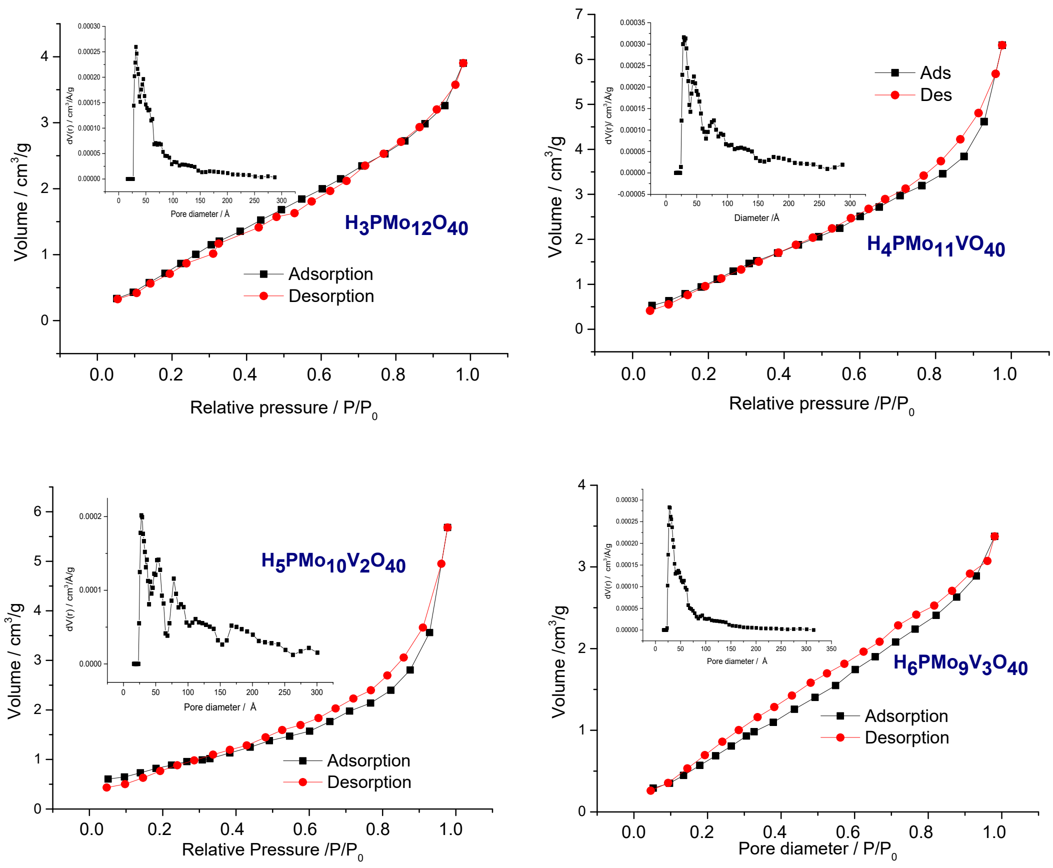

Figure 1SM Isotherms of adsorption and desorption of N_2 and volume and diameters porous (inset) of a pure phosphomolybdic acid catalyst and after the Vanadium doping.

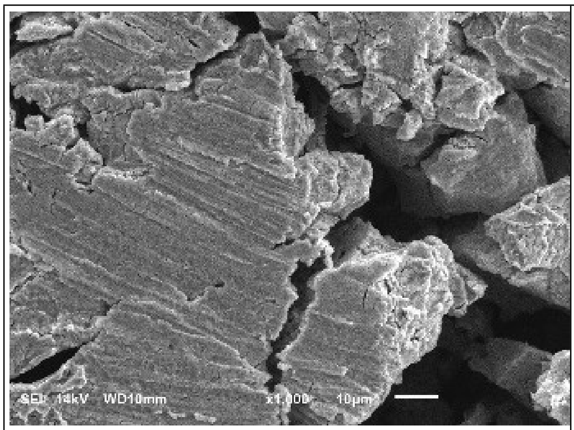

(a) $H_3PMo_{12}O_{40}$

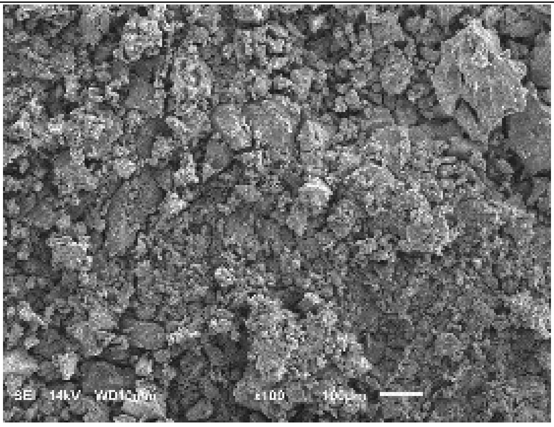

(b) $H_4PMo_{11}VO_{40}$

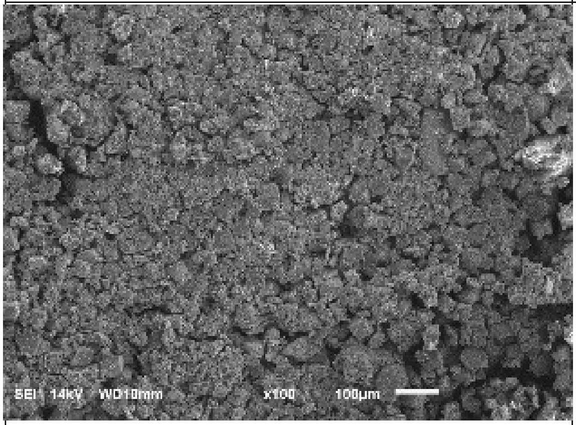

(c) $H_5PMo_{10}V_2O_{40}$

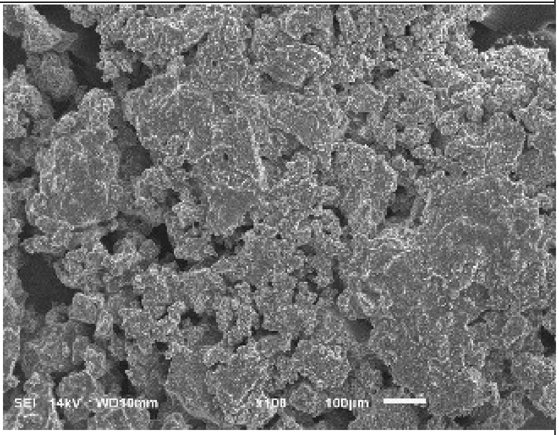

(d) $H_6PMo_9V_3O_{40}$

Figure 2SM. Scanning electronic microscopy images of undoped and Vanadium-doped phosphomolybdic acids.

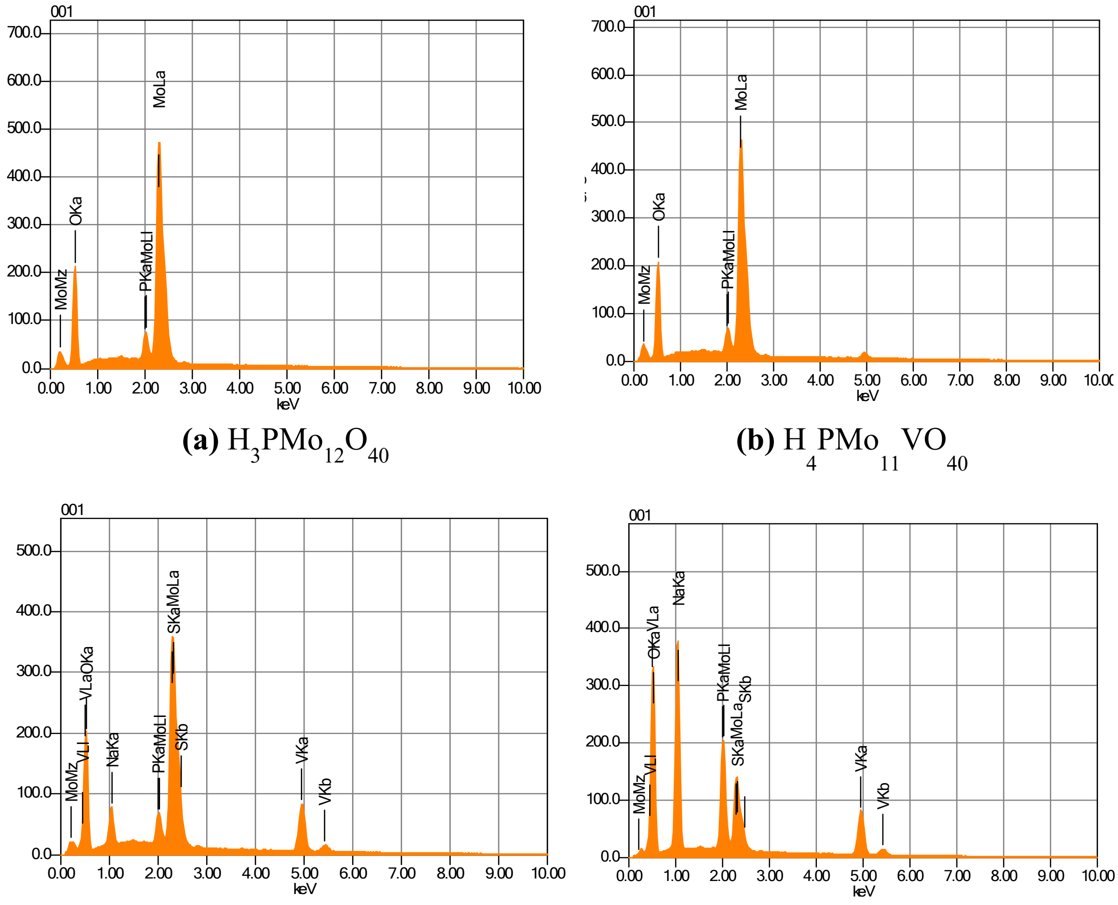

(a) $H_3PMo_{12}O_{40}$ **(b)** $H_4PMo_{11}VO_{40}$

(c) $H_5PMo_{10}V_2O_{40}$ **(d)** $H_6PMo_9V_3O_{40}$

Figure 3SM. EDS spectra of undoped and Vanadium-doped phosphomolybdate acids.

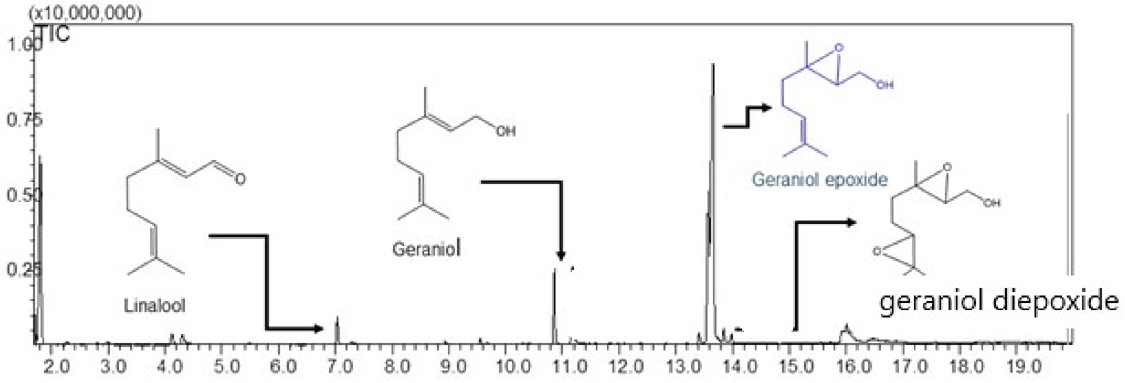

Figure 4SM. Typical chromatogram of oxidation reaction with hydrogen peroxide

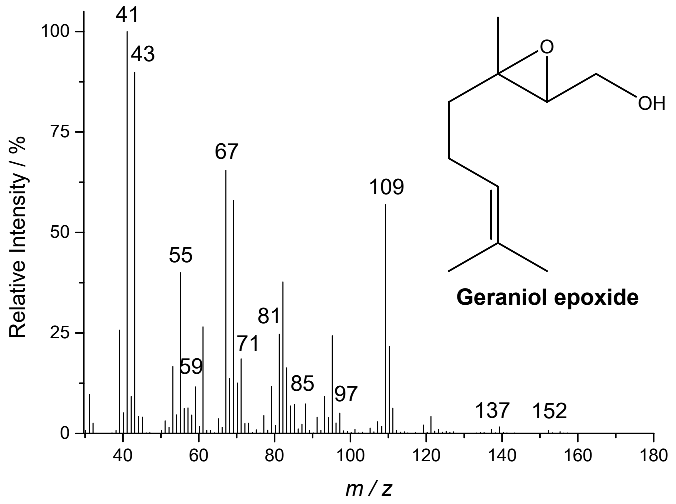

Figure 5SM. Fragmentogram of the geraniol epoxide

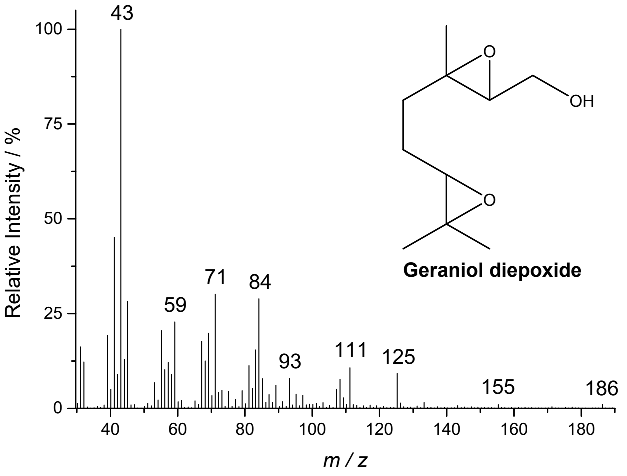

Figure 6SM1: Fragmentogram of the geraniol diepoxide

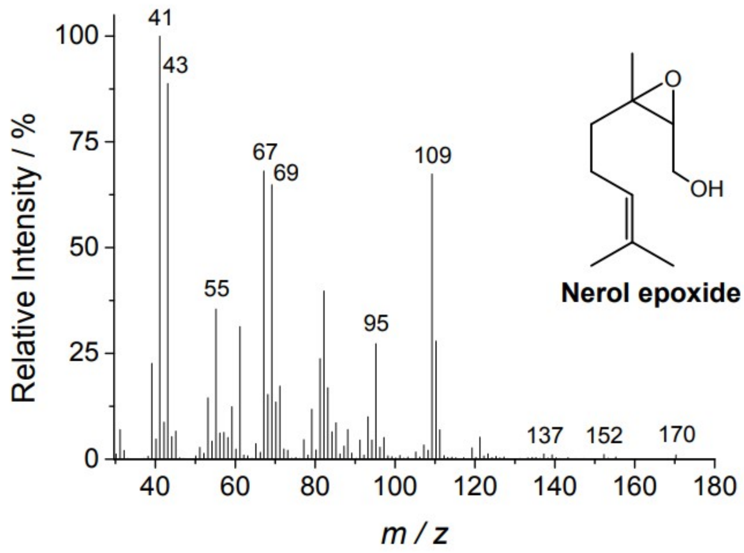

Figure 7SM1: Fragmentogram of the nerol epoxide

Table 1SM. Porosimetry characteristics of pure and Vanadium doped-Sodium phosphomolybdate salts[a]

Catalyst	S_{BET} (m^2/g)	V_{DFT} (cm^3/g)	D (Å)
$H_3PMo_{12}O_{40}$	1.4	1.7×10^{-3}	37.9
$H_4PMo_{11}VO_{40}$	2.7	8.2×10^{-3}	29.0
$H_5PMo_{10}V_2O_{40}$	2.0	7.2×10^{-3}	27.7
$H_6PMo_9V_3O_{40}$	1.9	4.5×10^{-3}	27.7

[a]S_{BET} = surface area; V_{DFT} = cumulative pore volume; D = pore diameter

Table 2SM. Hydration water number per mol of catalyst determined through thermal analysis.

Catalyst	Total hydration water (573 K)
$H_3PMo_{12}O_{40}$	6
$H_4PMo_{11}VO_{40}$	8
$H_5PMo_{10}V_2O_{40}$	5
$H_6PMo_9V_3O_{40}$	6

Table 5SM. Effect of the catalyst on the constant rate and TON of geraniol oxidation reactions with H_2O_2[a]

Catalyst	Rate constant[b] mmol/s	TON[c]
$H_3PMo_{12}O_{40}$	3.8×10^{-2}	143
$H_4PMo_{11}VO_{40}$	3.2×10^{-2}	143
$H_5PMo_{10}V_2O_{40}$	2.7×10^{-2}	119
$H_6PMo_9V_3O_{40}$	1.5×10^{-2}	104

[a]Reaction conditions: geraniol (2.75 mmol), H_2O_2 (2.75 mmol), toluene (internal standard), temperature (333 K), CH_3CN (10 mL).

[b]Rate constant: measured after 1 h reaction; [c]TON: measured after 8 h reaction

Table 6SM. Effect of $H_4PMo_{11}VO_{40}$ catalyst load on the constant rate and TON of geraniol oxidation reactions with H_2O_2[a]

Load Mol %	Rate constant[b] mmol/s x 10^{-4}	TON[c]
0.66	6.88	157
0.33	5.19	267
0.16	4.58	466
0.08	4.42	935
0.04	4.05	1738

[a]Reaction conditions: geraniol (2.75 mmol), H_2O_2 (2.75 mmol), toluene (internal standard), temperature (333 K), CH_3CN (10 mL).

[b]Rate constant: measured after 1 h reaction; [c]TON: measured after 8 h reaction
